# Incidence of atrial fibrillation and its association with long-term outcome in patients with an accessory pathway

**DOI:** 10.3389/fcvm.2025.1639305

**Published:** 2025-09-04

**Authors:** Gesa von Olshausen, Jonas Schwieler, Hamid Bastani, Tara Bourke, Ott Saluveer, Nikola Drca, Astrid Paul-Nordin, Emmanouil Charitakis, Fahd Asaad, Serkan Saygi, Yusuf Turkmen, Emma Svennberg, Finn Åkerström, Mats Jensen-Urstad, Frieder Braunschweig

**Affiliations:** ^1^ Department of Cardiology, Karolinska University Hospital, Stockholm, Sweden; ^2^ Medical Department I (Cardiology, Angiology, Pneumology), Klinikum Rechts der Isar, Technical University of Munich, Munich, Germany; ^3^ Heart and Lung Disease Unit, Department of Medicine, Karolinska Institutet, Stockholm, Sweden

**Keywords:** accessory pathway, atrial fibrillation, catheter ablation, catheter ablation registry, recurrence

## Abstract

**Objective:**

This study aimed to examine the incidence of atrial fibrillation in patients with an accessory pathway (AP) and its association with transient ischemic attack (TIA)/stroke as well as mortality over long-term follow-up.

**Methods:**

A total of 1,302 consecutive patients who underwent first-time ablation AP between 2005 and 2018 were included from the Karolinska Ablation Registry and followed up through the National Patient Registry and Cause of Death Registry.

**Results:**

Patients were 41.7 ± 15.7 years old and 60.9% were men. New-onset or recurrence of atrial fibrillation occurred in 111 patients (8.5%) after a follow-up of up to 10 years (median 6.8 years; interquartile range 3.9–9.9 years). Multivariable analysis revealed that a history of atrial fibrillation, hyperlipidemia, a higher BMI, and older age were independently associated with new-onset or recurrence of atrial fibrillation during follow-up. All-cause mortality and TIA/stroke occurred in 35 patients (2.7%) and 28 patients (2.2%) after a follow-up of 10 years, respectively. Multivariable analysis revealed that the occurrence of atrial fibrillation during follow-up was independently associated with both outcomes.

**Conclusion:**

In this large patient cohort with ablated APs, long-term follow-up revealed a high incidence of atrial fibrillation with 8.5%. Occurrence of atrial fibrillation during follow-up was independently associated with both all-cause mortality and TIA/stroke. Hence, closer monitoring for atrial fibrillation is advisable in patients with ablated APs, especially in those with a prior history of atrial fibrillation.

## Introduction

Atrioventricular reentrant tachycardia (AVRT) is a common type of supraventricular tachycardia characterized by an accessory pathway (AP) that allows direct electrical conduction between the atria and ventricles. This AP allows formation of a reentrant circuit involving the AV-node and, thus, provides a substrate for recurrent episodes of rapid tachycardia. Most patients with APs have ventricular preexcitation with a characteristic delta wave in the resting 12-lead ECG during sinus rhythm, but in a substantial proportion preexcitation is absent (“concealed conduction”). Those with preexcitation and clinical AVRT are diagnosed with Wolff–Parkinson–White syndrome. More men than women are affected by APs (1).

Catheter ablation of AP has become the treatment of choice for symptomatic and recurrent AVRT with a high rate of success and few complications (2). Furthermore, invasive electrophysiology for risk assessment (and ablation if appropriate) is also recommended in asymptomatic patients with preexcitation who have a small but sizable risk of sudden death (3).

Among patients with AP, the incidence of atrial fibrillation has been reported ranging from 9%–24% (4–7) which is higher compared to the general adult population (around 3%) (8). In general, atrial fibrillation is associated with an increased risk of all-cause mortality and morbidity including stroke. In patients with an antegrade conduction over the AP, atrial fibrillation may lead to rapid ventricular response potentially degenerating into ventricular fibrillation causing cardiac arrest and death (9).

Therefore, assessing the risk of atrial fibrillation in patients with an AP is essential to provide early diagnosis and treatment. However, there is limited data from systematic analyses evaluating the incidence of atrial fibrillation in patients with an ablated AP in a large patient cohort with long-term follow-up. This study aimed to examine the incidence and risk factors of atrial fibrillation in patients with an ablated AP. Furthermore, the association of atrial fibrillation during follow-up with transient ischemic attack (TIA)/stroke and all-cause mortality was investigated.

## Methods

### Study protocol and setting

All patients who underwent electrophysiology procedures at Karolinska University Hospital from January 2005 to September 2018 were included in the analysis. Relevant patient characteristics and procedural details were prospectively gathered during the ablation procedure and documented in a computerized database.

The National Patient Registry and the Cause of Death Registry, managed by the Swedish Board of Health and Welfare, supplied data on the date and cause of death, additional baseline comorbidities and the cause-specific hospitalizations, defined based on ICD-10 codes. Each patient was uniquely identified using the personal identification number assigned to all permanent Swedish citizens, enabling the integration of data from various registries. The establishment of this analysis and the linkage of the aforementioned registries were approved by the Swedish Ethical Review Authority (etikprövningsmyndigheten; diarienummer: 2019-00086) and conducted in accordance with the Declaration of Helsinki. According to the approval for this study, individual patient consent was not required.

### Study cohort and post-ablation follow-up

A cohort of patients undergoing AP ablation or electrophysiology procedure for risk assessment of AP was selected to ensure a reliable diagnosis of APs. Consecutive patients (≥18 years old at the time of index procedure) undergoing first-time catheter ablation of AP from 01 January 2005 to 30 September 2018 were enrolled. History of atrial fibrillation at baseline was defined as atrial fibrillation within 6 years prior to index procedure. Catheter ablations of AP were performed according to conventional and local standards, as described previously (10, 11). All patients were followed from the time of their index procedure until the date of death, emigration, or the end of the study (31st December 2018).

### Study outcomes

Study outcomes were defined as new-onset or recurrence of atrial fibrillation, all-cause mortality and TIA/stroke. Atrial fibrillation and TIA/stroke were diagnosed during both inpatient and outpatient visits. The definitions of the variables used in this study are provided in Supplementary Table S1.

### Statistical analysis

All continuous variables are presented as means ± standard deviation or medians with interquartile range (IQR). Categorical variables are reported as frequencies and percentages. Clinical outcomes were assessed over a ten-year period which was considered a meaningful duration for reflecting long-term follow-up. Univariate and multivariable backward logistic regression analyses were conducted to identify factors associated with the new onset or recurrence of atrial fibrillation, all-cause mortality, and hospitalization for TIA/stroke. The multivariable model included factors with a *p*-value <0.05 in the univariate analysis. Since all available AP patients were included, no sample size calculation was performed. All statistical tests and confidence intervals were two-sided, with a significance level of 0.05. Statistical analyses were performed using SPSS software, version 29 (IBM Corp., Armonk, New York).

## Results

### Baseline characteristics and index procedure details of patients undergoing first-time catheter ablation for AP

From January 1, 2005, to September 30, 2018, a total of 16,417 invasive electrophysiological procedures were performed in 12,247 patients. From this patient cohort, 1,302 distinct patients with APs met the inclusion criteria and were included in the analysis (Supplementary Figure S1). Their mean age was 41.7 ± 15.7 years and 793 patients (60.9%) were men. At baseline, 116 patients (8.9%) had a prior diagnosis of atrial fibrillation. Table 1 presents all baseline clinical characteristics of the study participants. Table 2 outlines the characteristics related to the index procedure.

**Table 1:** Baseline characteristics of patients with catheter ablation for AP.

Baseline characteristics	AP patients (*n* = 1,302)
Age (years)	41.7 ± 15.7
Men, *n* (%)	793 (60.9)
BMI^a^	25.3 ± 5.3
Ischemic heart disease, *n* (%)	38 (2.9)
Heart failure, *n* (%)	17 (1.3)
Arterial hypertension, *n* (%)	179 (13.7)
Diabetes mellitus, *n* (%)	38 (2.9)
Hyperlipidemia, *n* (%)	52 (4.0)
History of TIA/stroke, *n* (%)	19 (1.5)
CHA2DS2-VA-Score (points)	0.31 ± 0.72
History of atrial fibrillation, *n* (%)	116 (8.9)

AP, accessory pathway; BMI, body-mass-index; TIA, transient ischemic attack.

^a^ Data available in 66.1% of all patients.

**Table 2:** Index procedure related characteristics of patients with catheter ablation for AP.

Baseline characteristics	AP patients (*n* = 1,302)
Symptoms before index procedure, *n* (%)	
Palpitations	1,104 (84.8)
Syncope	77 (5.9)
Dizziness	36 (2.8)
None	84 (6.5)
Energy delivery, *n* (%)	
RF-energy	1,171 (89.9)
Not specified	131 (10.1)
Fluoroscopy time (min)	16.0 (IQR: 9.5; 26.2)
Procedure time (min)	115.3 (IQR: 88.1; 157.4)
AP location, *n* (%)	
Free-wall AP	820 (63.0)
Septal AP	349 (26.8)
Not specified	133 (10.2)
AP, *n* (%)	
Concealed	546 (41.9)
Overt	744 (57.1)
Not specified	12 (0.9)

AP, accessory pathway; RF, radiofrequency; IQR, interquartile range.

The mean duration of follow-up was 6.9 ± 3.7 years (median 6.8 years; interquartile range 3.9–9.9 years).

### Outcomes: atrial fibrillation, all-cause mortality and TIA/stroke

**Figure 1:**
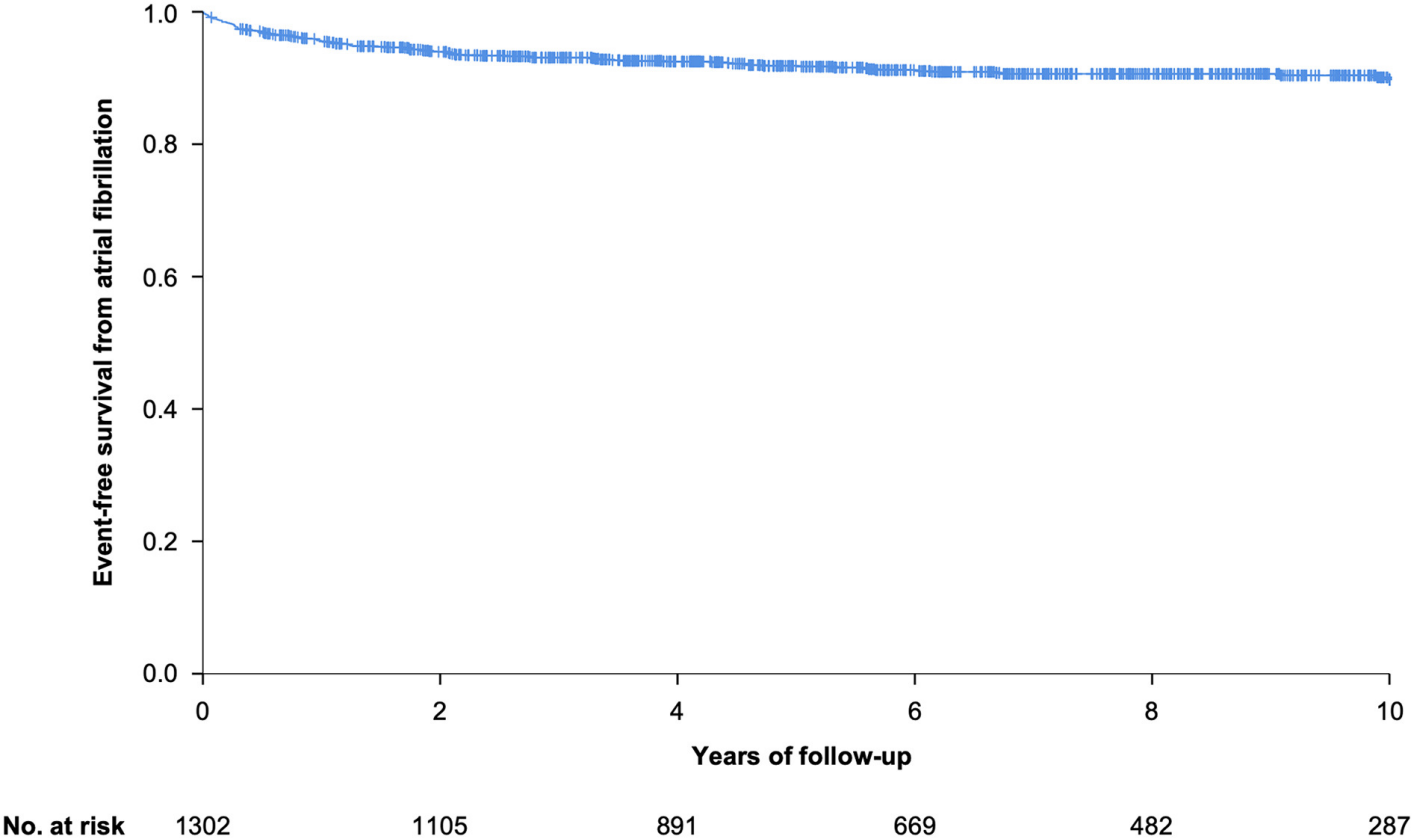
Kaplan–Meier analysis of event-free survival from atrial fibrillation after 10 year follow-up.

New-onset or recurrence of atrial fibrillation occurred in 111 patients (8.5%) during follow-up of up to 10 years. The corresponding Kaplan–Meier-curve is presented in Figure 1. The mean period from AP ablation to diagnosis of new-onset or recurrence of atrial fibrillation was 1.8 ± 2.2 years. Excluding patients with a history of atrial fibrillation, new-onset of atrial fibrillation occurred in 54 patients (4.6%). Among patients with prior history of atrial fibrillation at baseline, recurrence of atrial fibrillation was observed in 57 patients (49.1%). History of atrial fibrillation, hyperlipidemia, a higher BMI and older age were independently associated with new-onset or recurrence of atrial fibrillation across the entire patient cohort in multivariable analysis (Supplementary Table S2). Table 3 and Supplementary Figure S2, supplementary, present the prevalence of atrial fibrillation based on age at the end of follow-up.

**Table 3:** Prevalence of atrial fibrillation according to age at the end of follow-up.

Age group	All AP patients (*n* = 1,302) %
<60 years (*n* = 892)	5.8
60–69 years (*n* = 196)	8.7
70–79 years (*n* = 164)	13.4
≥80 years (*n* = 50)	40.0
All ages	8.5

AP, accessory pathway.

All-cause mortality occurred in 35 patients (2.7%) and cardiovascular mortality in 9 (0.7%) after a follow-up of 10 years. Diabetes mellitus, occurrence of atrial fibrillation during follow-up, arterial hypertension and older age were independently associated with all-cause mortality in multivariable analysis (Supplementary Table S3).

TIA/stroke occurred in 28 patients (2.2%) after a follow-up of 10 years. Occurrence of atrial fibrillation during follow-up, history of TIA/stroke and older age were independently associated with TIA/stroke in multivariable analysis (Supplementary Table S4).

## Discussion

In this large patient cohort with ablated APs, long-term follow-up revealed a high incidence of atrial fibrillation with 8.5%. After adjusting for covariates, the occurrence of atrial fibrillation during follow-up continued to be independently associated with both all-cause mortality and TIA/stroke.

As far as we are aware, this is the largest patient cohort with ablated AP analyzed in terms of incidence of atrial fibrillation and its association with long-term outcome. The mean age with 41.7 ± 15.7 years at the time of AP ablation and higher proportion of male patients was comparable to previous studies (1, 6). The incidence of new-onset or recurrence of atrial fibrillation during follow-up was high with 8.5% compared to data from the general Swedish population (8) and at the lower range compared to previous data of AP patients (4–7). History of atrial fibrillation, hyperlipidemia, a higher BMI and older age remained independently associated with an increased risk of developing atrial fibrillation during follow-up, consistent with findings from previous studies (4, 7).

The prevalence of atrial fibrillation based on age at the end of follow-up increased with increasing age and was higher among all patient subgroups (<60 years: 5.8%; 60–69 years: 8.7%; 70–79 years: 13.4; ≥80 years: 40%) compared to Swedish general population-based data (<60 years: 0.6%; 60–69 years: 4.2%; 70–79 years: 9.7%; 80–89 years: 13.4%; ≥90 years: 9.0%) (8). Several mechanisms responsible for the genesis of atrial fibrillation in AP patients may be involved such as spontaneous degeneration of AVRT into atrial fibrillation, electrical properties of the AP, the effects of AP on atrial architecture, the intrinsic atrial muscle vulnerability (12, 13), the increased sympathetic activity and vagal withdrawal (14) and the influence of advancing age. Also the existence of a retrograde multiple or multifiber AP is related to atrial fibrillation inducibility (15). Several studies demonstrated a decrease of atrial fibrillation incidence after successful elimination of the AP, suggesting that the AP itself may play an important role in the initiation of atrial fibrillation at least in some patients (16). However, atrial fibrillation still occurs in other patients with AP even after successful ablation. This suggests that atrial vulnerability parameters can be modified through AP ablation in some patients while in others they persist despite ablation (15). In patients with recurrence of pre-existing atrial fibrillation, the natural course of atrial fibrillation also needs to be considered. In non-ablated populations, atrial fibrillation is known to progress over time - from paroxysmal to persistent forms, especially in the presence of structural heart disease or atrial remodeling (17). Thus, recurrence of atrial fibrillation in previously affected individuals may align with this course, and not necessarily be related to AP.

In the current study, nearly half the patients with history of atrial fibrillation had a recurrence of atrial fibrillation during follow-up after AP ablation (57 patients; 49.1%). The (retrospective) design of the current study does not allow to properly answer the question how and to which degree AP ablation impacts the occurrence of atrial fibrillation during follow-up. Nevertheless, due to the high rate of atrial fibrillation, continuation of anticoagulation therapy post AP ablation may be reconsidered in patients with prior atrial fibrillation and a relevant CHA2DS2-VA score.

Compared to our previous study of patients with atrioventricular nodal re-entrant tachycardia (AVNRT) (18), AP patients were younger, proportionally more male and had a lower comorbidity burden. This might explain the lower prevalence of atrial fibrillation at the end of follow-up in AP compared to AVNRT patients (but still being higher among all patient subgroups when compared to general population-based data).

As occurrence of atrial fibrillation during follow-up was independently associated with both all-cause mortality and TIA/stroke, closer clinical monitoring and follow-up in patients with ablated APs is advisable. This especially accounts for patients with prior atrial fibrillation underlining the importance of a close follow-up and a careful consideration before discontinuing anticoagulation in these patients.

## Limitations

All data were collected at a single electrophysiology unit, and the results may vary compared to those from other centers.

Follow-up after ablation for atrial fibrillation did not involve intensive monitoring (such as regular Holter-ECG, transtelephonic monitoring, or implantable loop recorders) but was instead based on patient presentations during inpatient or outpatient visits. As a result, asymptomatic cases of atrial fibrillation may have been missed. However, our cohort reflects real-world data with practical clinical relevance.

A potential misclassification between AVRT and atrial fibrillation cannot be ruled out, meaning that an AVRT may have been (mis)classified as atrial fibrillation, particularly before AVRT ablation and when patients were followed up by non-cardiologists.

Energy delivery has not been specified in around 10% of the AP patients. According to documentation most of these patients have still been ablated. Few of these patients might have only received an electrophysiology procedure with AP diagnosis including risk assessment.

Primarily patients with ablated AP were analyzed. Therefore, the results may differ for patients with suspected but not ablated APs, as AP ablation itself may influence the incidence of atrial fibrillation during follow-up.

The administration of all types of treatments for atrial fibrillation, including both medical and ablative therapies, was not included in this study.

## Conclusion

In this large patient cohort with ablated APs, long-term follow-up revealed a high incidence of atrial fibrillation with 8.5%. Occurrence of atrial fibrillation during follow-up was independently associated with both all-cause mortality and TIA/stroke. Hence, closer monitoring for atrial fibrillation is advisable in patients with ablated AP, especially in those with a prior history of atrial fibrillation.

## Data Availability

The raw data supporting the conclusions of this article will be made available by the authors, without undue reservation.
