# Supplementary figures and images for: Incidence of atrial fibrillation and its association with long-term outcome in patients with an accessory pathway
Source: Front Cardiovasc Med. 2025 Sep 4;12:1639305. doi: 10.3389/fcvm.2025.1639305 (PMC12443779; doi:10.3389/fcvm.2025.1639305)

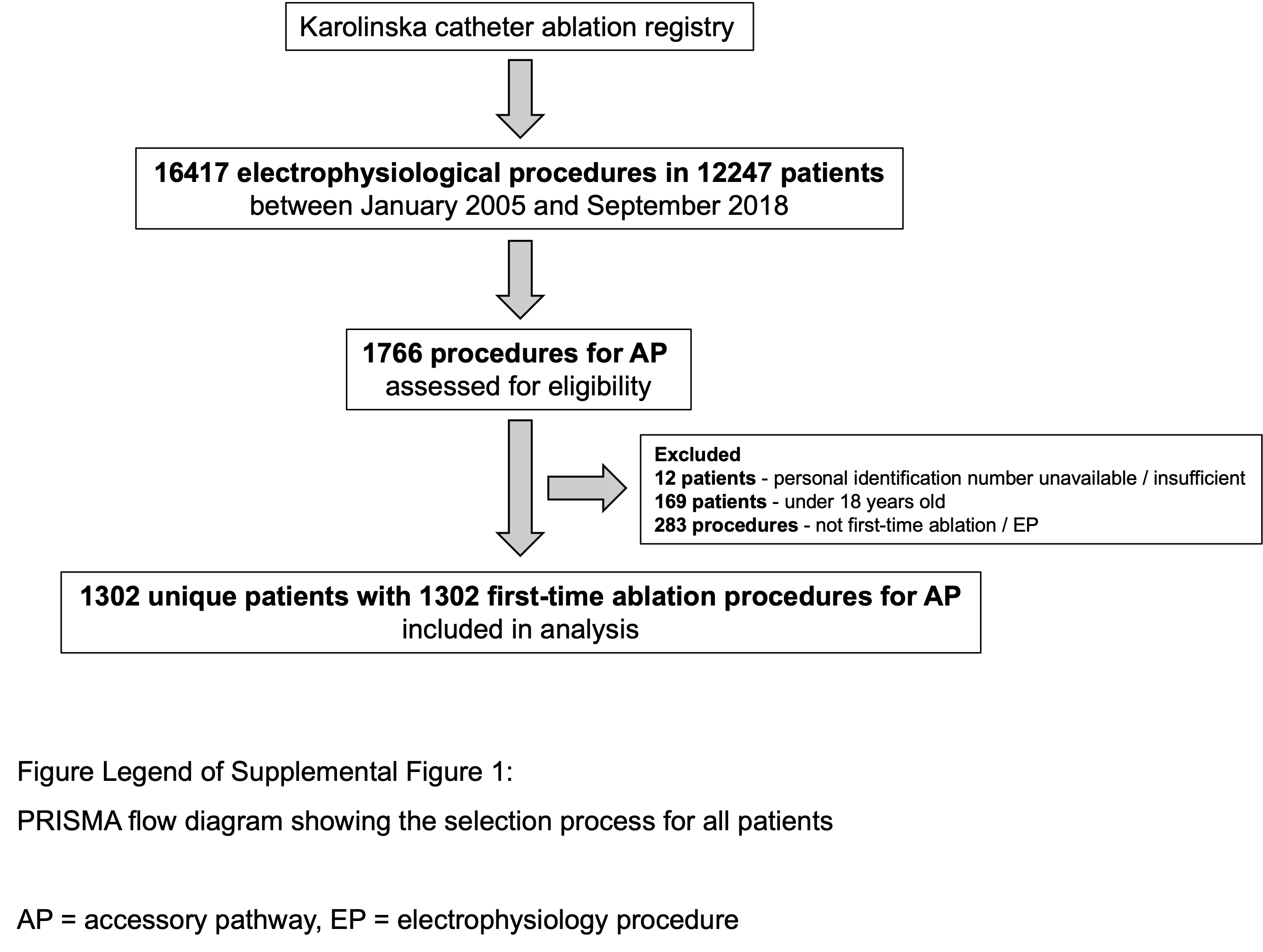

Supplement: Supplementary file 1 [file Image1.tiff]

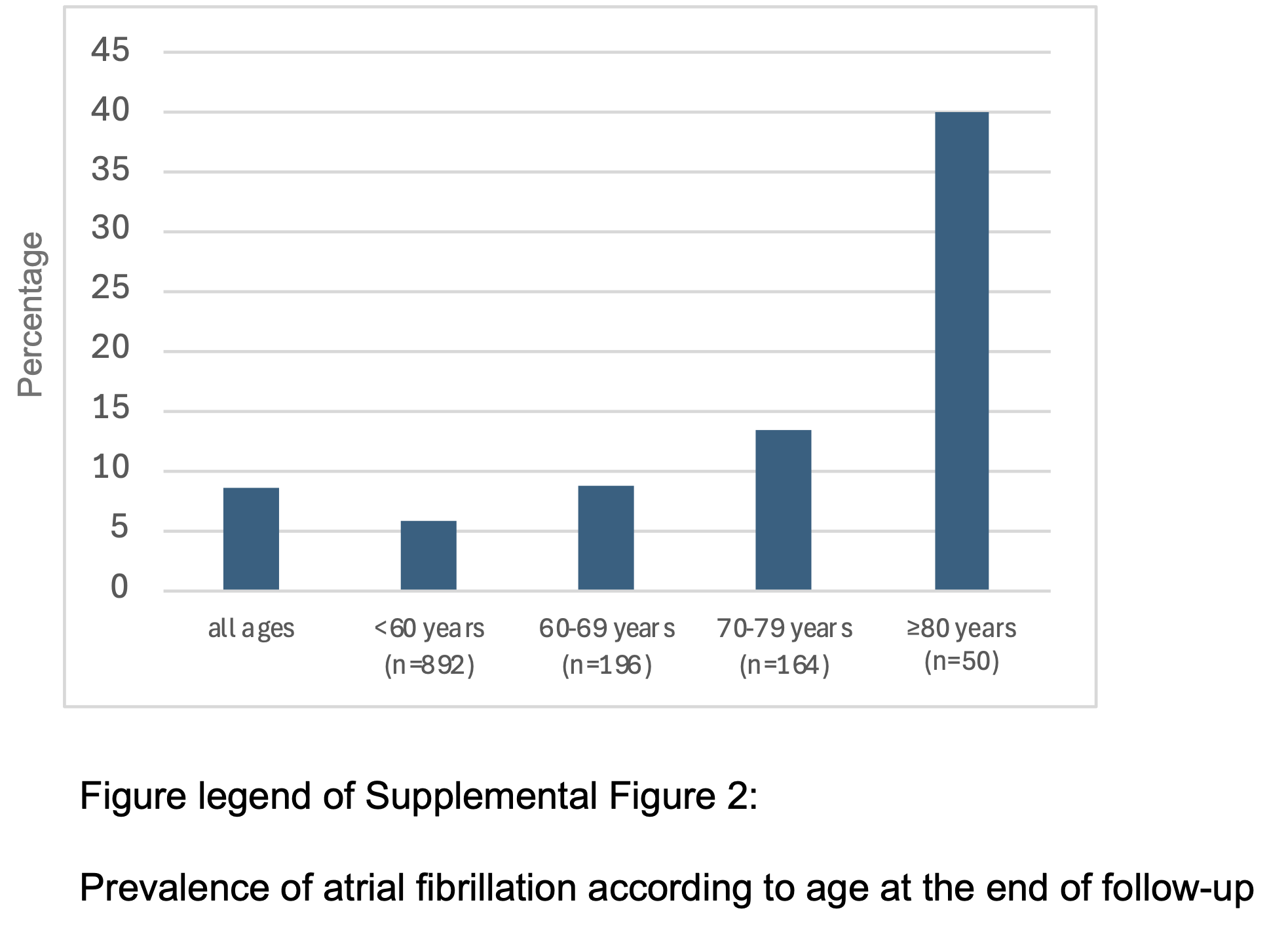

Supplement: Supplementary file 2 [file Image2.tiff]
