# Supplementary material for: Incidence of atrial fibrillation and its association with long-term outcome in patients with an accessory pathway
Source: Front Cardiovasc Med. 2025 Sep 4;12:1639305. doi: 10.3389/fcvm.2025.1639305 (PMC12443779; doi:10.3389/fcvm.2025.1639305)
Supplement: Supplementary file 3 [file Table1.docx]

Supplemental Table 1

Variable Definitions

| **Variable** | **Code** | **Registry** |
| --- | --- | --- |
| **Comorbidities** | | |
| **Heart failure** | ICD-10: I50 | NPR |
| **Arterial Hypertension** | ICD-10: I10-5 | NPR |
| **Diabetes** | ICD-10: E10-4 | NPR |
| **Hyperlipidemia** | ICD-10: E78 | NPR |
| **TIA /Stroke** | ICD-10: I60-4, I690-4, G45 | NPR |
| **Atrial fibrillation** | ICD-10: I48 | NPR |
| **Outcomes** | | |
| **Atrial fibrillation** | ICD-10: I48 | NPR |
| **TIA/Stroke** | ICD-10: I60-4, G45 | NPR |
| **All-cause death** | ICD-10: any | Cause of death |

ICD-10 codes were considered in any position for comorbidities and in primary (TIA/stroke) or primary/secondary position (atrial fibrillation) for outcomes

NPR = National Patient Registry, TIA = transient ischemic attack.

Supplemental Table 2

Logistic regression analysis for new-onset or recurrence of atrial fibrillation during 10 years of follow-up

| **Univariate analysis** | | |
| --- | --- | --- |
| **Variable** | **Odds ratio (95% CI)** | **p-value** |
| Age | 1.049 (1.036 – 1.063) | <0.001 |
| Sex, Female | 0.762 (0.505 – 1.149) | 0.195 |
| BMI | 1.083 (1.034 – 1.134) | <0.001 |
| Ischemic heart disease | 2.519 (1.083 – 5.860) | 0.032 |
| Heart failure | 7.949 (2.964 – 21.317) | <0.001 |
| Arterial hypertension | 3.348 (2.164 – 5.182) | <0.001 |
| Diabetes mellitus | 1.655 (0.633 – 4.329) | 0.304 |
| Hyperlipidemia | 4.379 (2.293 – 8.362) | <0.001 |
| History of TIA / stroke | 3.966 (1.401 – 11.223) | 0.009 |
| History of atrial fibrillation | 20.252 (12.849 – 31.922)  7 – 1.615) | <0.001 |
| Accessory pathway, overt (vs concealed) | 0.757 (0.511 – 1.122) | 0.165 |
| AP location septal (vs free-wall) | 0.906 (0.575 – 1.429) | 0.672 |
| **Multivariable analysis** | | |
| History of atrial fibrillation | 21.459 (11.586 – 39.747) | <0.001 |
| Hyperlipidemia | 2.774 (1.007 – 7.641) | 0.048 |
| BMI | 1.082 (1.005 – 1.123) | 0.032 |
| Age | 1.033 (1.013 – 1.055) | 0.002 |

CI = confidence interval, BMI = body-mass-index, TIA = transient ischemic attack, AP = accessory pathway

Supplemental Table 3

Logistic regression analysis for all-cause mortality during 10 years of follow-up

| **Univariate analysis** | | |
| --- | --- | --- |
| **Variable** | **Odds ratio (95% CI)** | **p-value** |
| Age | 1.086 (1.058 – 1.114) | <0.001 |
| Sex, Female | 0.708 (0.344 – 1.458) | 0.348 |
| BMI | 0.993 (0.906 – 1.089) | 0.881 |
| Ischemic heart disease | 4.679 (1.564 – 13.996) | 0.006 |
| Heart failure | 5.059 (1.112 – 23.022) | 0.036 |
| Arterial hypertension | 2.234 (1.029 – 4.849) | 0.042 |
| Diabetes mellitus | 9.968 (4.046 – 24.558) | <0.001 |
| Hyperlipidemia | 3.277 (1.112 – 9.654) | 0.031 |
| History of TIA / stroke | 4.456 (0.989 – 20.080) | 0.052 |
| History of atrial fibrillation | 2.176 (0.884 – 5.355)  7 – 1.615) | 0.091 |
| Atrial fibrillation during follow-up | 5.349 (2.546 – 11.236) | <0.001 |
| AP overt (vs concealed) | 0.772 (0.394 – 1.511) | 0.449 |
| AP location septal (vs free-wall) | 1.070 (0.501 – 2.284) | 0.861 |
| **Multivariable analysis** | | |
| Diabetes mellitus | 7.419 (2.763 – 19.925) | <0.001 |
| Atrial fibrillation during follow-up | 2.992 (1.299 – 6.889) | 0.010 |
| Arterial hypertension | 2.732 (1.078 – 6.925) | 0.034 |
| Age | 1.088 (1.056 – 1.120) | <0.001 |

CI = confidence interval, BMI = body-mass-index, TIA = transient ischemic attack, AP = accessory pathway

Supplemental Table 4

Logistic regression analysis for TIA/stroke during 10 years of follow-up

| **Univariate analysis** | | |
| --- | --- | --- |
| **Variable** | **Odds ratio (95% CI)** | **p-value** |
| Age | 1.087 (1.056 – 1.119) | <0.001 |
| Sex, Female | 0.733 (0.329 – 1.634) | 0.448 |
| BMI | 1.002 (0.904 – 1.111) | 0.969 |
| Ischemic heart disease | 6.078 (1.999 – 18.481) | 0.001 |
| Heart failure | 10.800 (2.919 – 39.958) | <0.001 |
| Arterial hypertension | 2.136 (0.894 – 5.099) | 0.088 |
| Diabetes mellitus | 2.645 (0.605 – 11.573) | 0.196 |
| Hyperlipidemia | 4.257 (1.421 – 12.751) | 0.010 |
| History of TIA / stroke | 13.989 (4.322 – 45.278) | <0.001 |
| History of atrial fibrillation | 5.166 (2.281 – 11.700)  7 – 1.615) | <0.001 |
| Atrial fibrillation during follow-up | 10.400 (4.812 – 22.478)  7 – 1.615) | <0.001 |
| AP overt (vs concealed) | 0.467 (0.217 – 1.005) | 0.052 |
| AP location septal (vs free-wall) | 0.581 (0.216 – 1.562) | 0.282 |
| **Multivariable analysis** | | |
| Atrial fibrillation during follow-up | 5.111 (2.211 – 11.819) | <0.001 |
| History of TIA / stroke | 4.358 (1.149 – 16.539) | 0.031 |
| Age | 1.066 (1.035 – 1.098) | <0.001 |

CI = confidence interval, BMI = body-mass-index, TIA = transient ischemic attack, AP = accessory pathway
